# Predatory Billing by a Concierge Home Health Agency Following Acute Hospitalization

**DOI:** 10.7759/cureus.112110

**Published:** 2026-07-05

**Authors:** Leela Rao, Jonah Swartzberg, Karen Galvez-Maquindang, Navid Darouian

**Affiliations:** 1 Medicine, Occidental College, Los Angeles, USA; 2 Medicine, University of California, Los Angeles, Los Angeles, USA; 3 Internal Medicine, University of California Los Angeles David Geffen School of Medicine, Los Angeles, USA; 4 Internal Medicine, University of California, Los Angeles, Los Angeles, USA

**Keywords:** cognitive vulnerability, concierge care, discharge planning, elder exploitation, elder financial abuse, geriatrics, home health

## Abstract

Elder financial exploitation is an underrecognized form of elder abuse that can occur in medical and post-acute care settings. We present the case of a 74-year-old man with multiple comorbidities who was hospitalized following a mechanical fall with head trauma and concussion. During his inpatient stay, while experiencing residual altered mental status, he was approached by a representative of a concierge home health agency who presented discharge requirements inconsistent with those of his clinical team, resulting in the patient signing a costly contract for caregiver services that substantially exceeded his documented clinical needs both in scope and expense. The patient's family, despite repeated attempts to engage with discharge planning, was excluded from the decision. The contract was later identified as financially exploitative; a replacement agency provided equivalent care at approximately one-seventh the cost. This case illustrates a reproducible pattern of predatory conduct targeting cognitively vulnerable hospitalized elders and highlights systemic gaps in hospital discharge safeguards.

## Introduction

It is estimated that at least 10% of adults over the age of 65 experience some form of elder abuse [1]. About 5.5% of older adults experience financial exploitation, which was recently recognized by the Centers for Disease Control (CDC) as a serious public health problem [2]. Frailty, or the accumulation of age-related health deficits and increased vulnerability to stressors, has been correlated with greater rates of financial exploitation, highlighting the importance of protective interventions for older hospitalized individuals [3]. The cognitive vulnerability associated with frailty can be taken advantage of in a healthcare setting, causing financial loss to patients and constituting fraud. It is estimated that between 3% and 10% of yearly total health care expenditures in the United States are lost to fraud, making this a significant concern [4].

We present a case involving a hospitalized older adult who, during a period of transient cognitive impairment following an acute injury, was approached by an outside agency and agreed to a costly service arrangement that was not consistent with the recommendations of his clinical team. The transition from hospital to home is often marked by time pressure, complex decision-making, and reduced family oversight, which may leave cognitively vulnerable patients susceptible to arrangements that do not reflect their actual care needs. This case raises concerns about the potential for financial elder abuse during such transitions and highlights a need for additional systemic safeguards to protect this population.

## Case presentation

**Figure 1:**
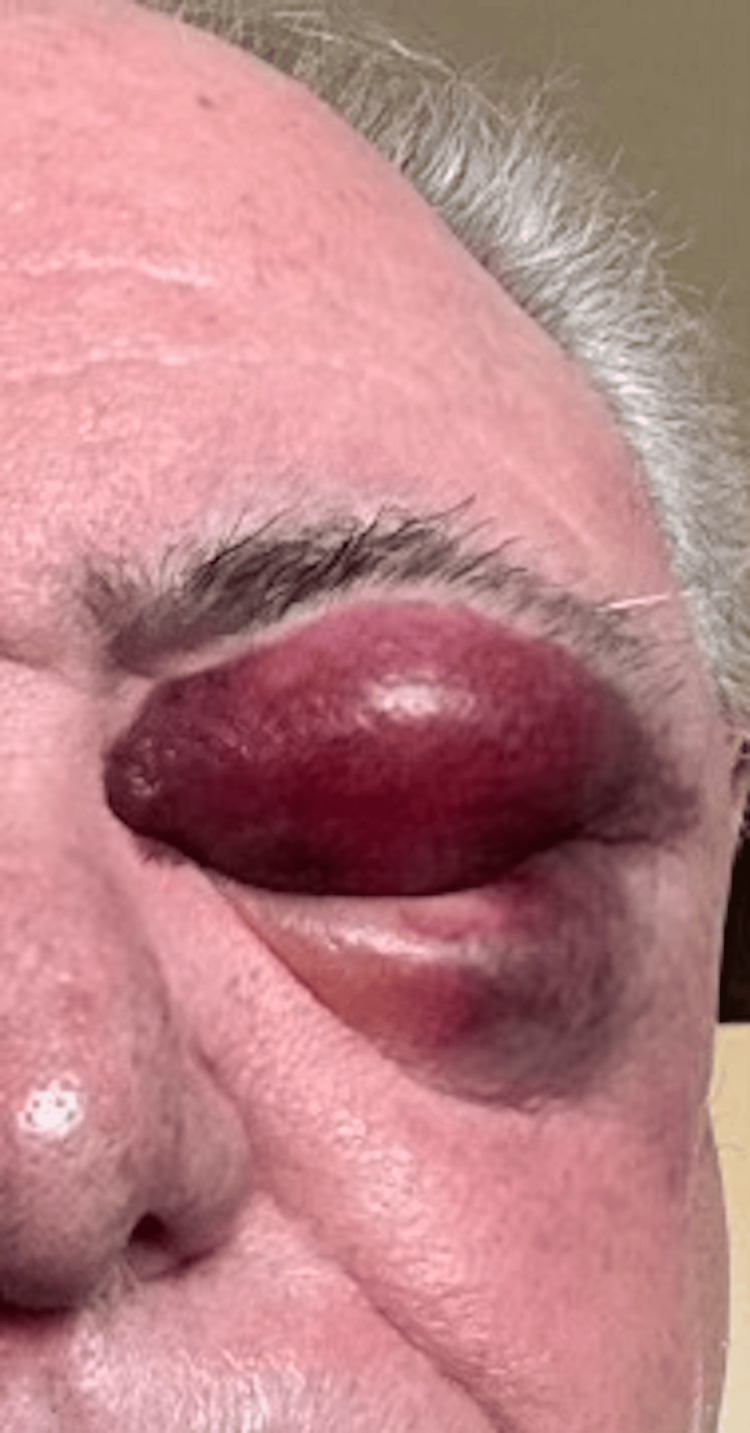
Left periorbital hematoma of a patient following a traumatic injury.

A 74-year-old man with a history of non-valvular atrial fibrillation on rivaroxaban (Xarelto) and a repaired ascending aortic aneurysm presented to the emergency department following a mechanical fall resulting in head trauma. On initial assessment, the patient demonstrated altered mental status with impaired recall of recent events. Physical examination was notable for a significant periorbital hematoma and facial soft-tissue injury as noted in Figure 1.

Neurological assessment revealed altered mental status and clinical features consistent with concussion, including disorientation, slowed processing, and diminished recall. Substantial gait instability was also documented. Given his anticoagulated state, neurological compromise, and fall risk, the patient was deemed unsafe for discharge and was admitted to an observation unit under medicine service.

The inpatient course was notable for the following: Trauma surgery evaluation on the day of arrival confirmed no need for surgical intervention for the periorbital and soft-tissue hematoma. Conservative management was pursued.

Pain was managed with intravenous analgesics, including opioids, after oral agents proved insufficient. The patient remained confused throughout the hospitalization, with persistent disorientation and impaired recall noted on serial assessments. The altered mental status was believed to be primarily related to the mild traumatic brain injury sustained during the fall, with the opioid analgesic regimen felt to be a contributing factor.

Physical and occupational therapy (PT/OT) evaluated the patient and determined that, with appropriate caregiver support, he was medically stable for discharge to home. Their clinical recommendation included caregiver assistance at the time of discharge, with a single caregiver deemed sufficient to meet his needs.

The patient's daughter, who had been in contact with the emergency department physician at presentation, was not included in discharge-related discussions during the hospitalization.

During the hospitalization, the patient was introduced to a representative of a concierge home health agency. The representative met with the patient privately at a time when he continued to exhibit altered mental status. During this meeting, the patient was informed that two 24-hour registered nurses would be required for discharge to proceed, a requirement that differed from the discharge plan recommended by the PT/OT team and attending physician, as well as from the discharge criteria documented by the hospital care team.

The patient, apparently acting on the belief that these services were mandatory for his release, signed a service contract with the concierge agency. The rate established under this contract was substantially higher than the cost of services typically required for a single caregiver. The patient's family was not informed of the agency's recommendations, which differed from those of the treating clinical team, nor were they made aware of the financial decisions made during a period of documented altered mental status. He was subsequently discharged without notification to his family.

Upon becoming aware of the discharge and the signed contract, the family immediately terminated the concierge agency’s services. A trusted regional home health provider, AccentCare (Dallas, Texas), was then contacted to independently assess the patient’s care needs. AccentCare met with the patient at his home and determined that a single caregiver, rather than two 24-hour registered nurses, appropriately addressed his clinical requirements. Their recommended service was engaged at approximately one-seventh the cost of the concierge agency’s contract, with full family participation in decision-making.

## Discussion

This case exemplifies several convergent features that constitute a recognizable pattern of elder financial exploitation in the post-acute discharge context.

Targeting of cognitive vulnerability

The patient was approached while experiencing altered mental status secondary to concussion, with documented disorientation, slowed processing, and poor recall. Cognitive impairment has been identified as a factor of vulnerability for exploitation, namely financial, because impaired executive function, memory, attention, and judgment may reduce an individual’s ability to recognize persuasive or misleading conduct [5]. In hospitalized older patients, decision-making capacity is also often impaired during acute brain or mind disorders. Research has shown that capacity assessments in hospitalized older adults are commonly performed in the context of discharge planning and that findings of incapacity are particularly common among patients with delirium or other cognitive impairments [6].

In this case, the patient signed a costly service contract during a period in which he continued to exhibit altered mental status, and the services contracted for were inconsistent with the clinical recommendations in place at the time. The encounter, therefore, did not occur in a period of stable, informed, and voluntary decision-making. It was a period during which heightened safeguards and family involvement were clinically warranted [6].

Misrepresentation of medical and institutional requirements

PT/OT had recommended a single caregiver at discharge based on the patient's assessed needs; this recommendation did not constitute a mandatory discharge requirement. The agency's representation that two 24-hour registered nurses were required for discharge was inconsistent with this clinical recommendation. Discharge decisions are made by the treating clinical team rather than by external agencies.

Additionally, the PT/OT, as well as the trusted service AccentCare (Dallas, Texas), specified a “caregiver,” not a registered nurse. Skilled home health care, such as a 24-hour registered nurse, is medically distinct and reserved for complex or unstable conditions requiring licensed clinical intervention, whereas caregiver services address daily custodial needs such as mobility and toileting [7]. Therefore, the behavior on behalf of the home health agency not only constituted misrepresentation of institutional requirements but also of medical necessity.

Gaps in family and surrogate engagement during discharge planning

Despite the daughter's involvement in the patient's clinical care throughout the hospitalization, she was not included in discharge planning. Isolation from relatives is cited as frequently being the first stage of financial exploitation, as it reduces external oversight [8]. This patient reportedly believed that the clinically unnecessary services offered by the concierge agency were required for discharge, reflecting a broader pattern in which vulnerable patients are persuaded to make financial decisions under misleading circumstances [8].

Gross financial disparity as an indicator of exploitation

The concierge agency’s rate was approximately seven times higher than that of a licensed community-based alternative providing equivalent or superior services. While premium pricing alone does not constitute abuse, a substantial disparity from market rate for services exceeding the level of care recommended by the clinical team may warrant further scrutiny. The subsequent independent assessment, conducted with full family involvement and arriving at materially different recommendations, supports the conclusion that the original contract did not reflect the patient's clinical needs.

Recommendations for clinical practice

Based on this case, several considerations may be relevant to discharge planning for cognitively vulnerable patients. Patients with cognitive impairment, recent concussion, or altered mental status may benefit from additional safeguards during discharge, including family or surrogate involvement in decisions with significant financial implications. Institutions may also wish to review policies regarding external agency access to inpatients and consider mechanisms to ensure that any discharge-related information communicated by outside agencies aligns with the clinical team's plan. Facilitating documented family involvement in discharge planning, when desired by the patient, may further support discharge safety. Finally, clinicians should remain aware of their reporting obligations regarding suspected elder financial exploitation, consistent with applicable state requirements.

## Conclusions

This case highlights how acute hospitalization and subsequent discharge planning can create a setting in which cognitively vulnerable older patients are exposed to financial exploitation. The patient was approached while experiencing a concussion that altered his mental status, and information about discharge requirements that differed from the clinical team's recommendations led him to sign an expensive contract for services that exceeded his documented clinical needs. Family was not involved in discharge planning, which removed an additional safeguard against inappropriate decision-making.

While this report describes a single case, the pattern observed may have broader clinical relevance and could merit further attention. Cognitive impairment, high-pressure discharge transitions, unregulated vendor access, and lack of family involvement may converge to create conditions that increase vulnerability to financial elder abuse. Hospitals and clinicians should recognize financial exploitation as a potential patient safety concern, particularly when older adults with altered mental status are asked to make substantial financial decisions during hospitalization. Strengthened discharge safeguards, clearer oversight of third parties, and inclusion of families to help make clear decisions may help prevent similar cases of exploitation in medically vulnerable patients.
